# Functional constraints channel mandible shape ontogenies in rodents

**DOI:** 10.1098/rsos.220352

**Published:** 2022-10-19

**Authors:** Morgane Dubied, Sophie Montuire, Nicolas Navarro

**Affiliations:** ^1^ Biogéosciences, UMR 6282 CNRS, EPHE, Université Bourgogne Franche-Comté, 6 bd Gabriel, 21000 Dijon, France; ^2^ EPHE, PSL University, 75014 Paris, France

**Keywords:** geometric morphometrics, mandible shape, ontogeny, rodent

## Abstract

In mammals, postnatal growth plays an essential role in the acquisition of the adult shape. During this period, the mandible undergoes many changing functional constraints, leading to spatialization of bone formation and remodelling to accommodate various dietary and behavioural changes. The interactions between the bone, muscles and teeth drive this developmental plasticity, which, in turn, could lead to convergences in the developmental processes constraining the directionality of ontogenies, their evolution and thus the adult shape variation. To test the importance of the interactions between tissues in shaping the ontogenetic trajectories, we compared the mandible shape at five postnatal stages on three rodents: the house mouse, the Mongolian gerbil and the golden hamster, using geometric morphometrics. After an early shape differentiation, by both longer gestation and allometric scaling in gerbils or early divergence of postnatal ontogeny in hamsters in comparison with the mouse, the ontogenetic trajectories appear more similar around weaning. The changes in muscle load associated with new food processing and new behaviours at weaning seem to impose similar physical constraints on the mandible, driving the convergences of the ontogeny at that stage despite an early anatomical differentiation. Nonetheless, mice present a rather different timing compared with gerbils or hamsters.

## 1. Introduction

Variation in time and space of developmental processes within an anatomical context will influence the mapping from genotypes to phenotypes [1–5]. Through ontogeny, mechanical forces in relation to cell properties are fundamental in shaping and remodelling tissues [6–9]. The physical constraints experienced by a growing organism could be similar across a certain range of anatomical contexts, and then may structure in a comparable way the phenotypic variation in influencing the spatialization of the underlying developmental processes. For example, during mammalian postnatal growth, major biomechanical changes in response to diet shift at weaning [10] influence the spatialization of bone remodelling [11–13]. Indeed, changes in muscle strain led to convergences in the developmental processes imposing some directionality in the ontogenetic trajectories despite a relatively diversified anatomical context [14], and constraining, as a consequence, the evolution of ontogenies and the adult shape variation [15]. However, early shape differentiation may have a profound effect on the directionality and intensity of shape changes through ontogenies. For instance, early functional requirements of the jaw or the forelimbs in relation to precocious suckling or crawling in marsupials have constrained the rate of shape evolution and disparity in comparison with eutherians [16–19].

The rodent mandible undergoes major modifications on its posterior part in order to accommodate the growth of masticatory muscles during the postnatal development [20]. These muscle–bone interactions constrain the main direction of shape changes in the mandible ontogeny across a wide range of rodent species [14,21]. Several functional and behavioural factors could affect one or the other component of these interactions. For example, suckling, gnawing and chewing movements have different functional requirements [22] and different diets will imply a different distribution of stresses [23]. Behaviours in relation to siblings or conspecifics (fights for example, [24]) will also have functional demands on the jaws through biting [25]. Changes in the timing and rates of these physical stresses should therefore be associated with shape changes through their modulation by muscle–bone interactions. This mechanism is well studied on adult shape covariation with changes in muscle anatomy and diet [26–28] or in context of ecological radiation [29,30], and it appears to lead to indirect responses of the mandible shape to the various sources of natural and sexual selections [31,32]. Much less is known on how this mechanism constrains ontogeny. A good model system for testing the importance of these interactions in shaping the ontogenies is the major functional shift affecting the mechanical stresses arising at weaning, which recomposes the field of physical constraints applied to the mandible in relation to the changes in muscle load associated with new food processing as well as new behaviours.

In this study, we focus on three rodents belonging to the suborder Myomorpha (the house mouse, Mongolian gerbil and golden hamster), and using geometric morphometrics, we compared the ontogenetic trajectories of the mandible shape at five postnatal stages. In Myomorpha, weaning is early and abrupt, as it occurs over a very short period of time, forcing the mandible to adapt rapidly to the chewing movement. If functional constraints arising at weaning channel the developmental trajectories, changes affecting the shape ontogenies should be similar across the three species despite their early shape divergence. Otherwise, shape differentiation inherited from differences of gestational length or pre-weaning divergence of ontogenies could provide enough variation in the anatomical context to condition the effect of functional constraints in different ways.

## 2. Material and methods

### 2.1. Breeding design and three-dimensional imaging

The specimens were bred in the central animal facility of the University of Burgundy (Project APAFIS#18405-2019011014262528). Six gravid females of mice (*Mus musculus*) were obtained from an internal breeding colony of Balb/c inbred strain at the University of Burgundy. Six gravid females of golden hamsters (*Mesocricetus auratus*) were obtained from the inbred colony of Janvier labs (RjHan:AURA). Gerbils (*Meriones unguiculatus*) were bred from breeding pairs (five females and two males) obtained from Charles River (RjTub:MON). In this study, at least one specimen per family was sacrificed at 7, 14, 35 and 63 days. The 100-day stage is represented by the mothers and, for gerbils, also by the fathers. Skeletons were cleaned using dermestid beetles. After removing broken specimens, 88 mandibles were scanned by µCT scan (Bruker Skyscan 1174) and reconstructed using Avizo® 9.2 (FEI systems). Before landmark digitization, three-dimensional models were decimated to 200 000 faces, using the Rvcg R package 0.18 [33]. Six specimens per species in average represent each stage.

### 2.2. Three-dimensional landmarks and semilandmarks collection

Fourteen landmarks were digitized, together with 22 curve semilandmarks along five distinct curves (with two semilandmarks on the coronoid process curve, three along the two lunar notch curves, seven on the curve between postcondylar and angular processes, and 10 on the masseter ridge; electronic supplementary material, figure S1). Digitization was done within the Digit3DLand R package 0.1.3 [34]. An atlas was created with five patches of surface semilandmarks totalizing 256 semilandmarks, from a mouse specimen with the PseudoLMGenerator module of the SlicerMorph package [35] on 3D Slicer 4.11 [36]. This atlas was transferred on all models using the ProjectSemiLM module. Curve and surface semilandmarks were then slid along their tangent by minimizing the bending energy and back-projected on the three-dimensional surfaces [37] using the Morpho R package 2.9 [33].

### 2.3. Statistical shape analysis

A full generalized Procrustes analysis (GPA) was performed using the Morpho R package [33]. Procrustes-aligned coordinates were projected on the tangent space at the mean shape and a principal component analysis was performed. Postnatal developmental trajectories were modelled from the expected marginal means of each developmental stage per species estimated from a multivariate linear model according to $\hat{z}=L\beta$, with L the design matrix corresponding to the linear contrasts for the different stages of species, and β, the least-square estimates of shape differences. Significance of the Procrustes sum of squares was evaluated using 1000 residual permutations with the RRPP R package 0.5.2 [38]. To check for allometry, an additional model with the effect of the log of the centroid size per species was estimated. The associated regression score [39], which corresponds to the most correlated shape variable to the allometric vectors, was computed.

Ontogenetic trajectories were parallel transported (PT) prior to analysis in order to remove the influences of inter-group shape differences on the shape changes along the trajectories [40]. To do so, and after checking if the variation is small enough, we used the linear shift algorithm, which provides a Euclidean approximation of the PT [41]. Briefly, as ontogenetic trajectories are defined based on discrete postnatal stages, we superimposed the 7-day-old expected marginal means $\left({\hat{z}}_{a=7}\right)$ of each species using a generalized Procrustes analysis without scaling to obtain a common reference $\left({\bar{z}}_{a=7}\right)$. We then superimposed the expected marginal means of all stages on this reference via ordinary Procrustes analysis using the deformetrics R package [42]. Finally, we added the tangent residual of each individual from the linear model to the transported mean of the group to which it belongs $\left({\hat{z}}_{a=j}^{\mathrm{tr}}\right)$ to obtain transported individual values. In other words, we translated the shapes into a common space, centred on the 7-day-old common mean shape. The least-square estimates of shape differences β were updated accordingly $\beta^{\mathrm{tr}}=\;L^{-1}{\hat{z}}^{\mathrm{tr}}$. Expected marginal means of growth vectors were then obtained from the linear contrast between two successive stages as $\hat{v}=(L_{a=j\;}-L_{a=j-1})\beta^{\mathrm{tr}}$.

### 2.4. Ontogenetic trajectory analysis

To assess the similarity of growth vectors between species, angles between a similar developmental stage *i* of two different species, *j* and *k*, were computed as $\theta =\;\cos^{-1}(v_{a=i,j}\cdot v_{\;a=i,k})$ with the growth vectors $\hat{v}\;$ normalized to unit length [43,44]. To assess whether the amount of shape changes between each stage was similar across species, the magnitude of the growth vector between two developmental stages was computed as the norm of the vector. Stepwise magnitude of the trajectories was computed as the sum of the vector norms, which represents the total shape change since birth. The magnitude of each step was further normalized by the growth length in days to allow comparison across growth periods. Approximation of standard errors on magnitudes and angles were computed based on the sampling distribution of $\beta^{\mathrm{tr}}$ [14,29,45], and according to $v^{*}∼\;N(\hat{v},S_{e}⊗(L_{a=j\;}-L_{a=j-1}){\left(X^{\prime }X\right)}^{-1}(L_{a=j\;}-L_{a=j-1}){\;}^{\prime })$, where $S_{e}$ is the residual covariance matrix, and ⊗  stands for the Kronecker product. Descriptive statistics on sampled angles were computed with the *circular* R package 0.4-93 [46]. Pairwise differences between trajectories in term of path distances, angles and shapes were evaluated using 1000 permutations [47].

The influence of weaning on the direction of growth was inferred by comparing the trajectory during weaning (14–35 days) with the trajectories immediately before (7–14 days) and after (35–63 days). These changes were computed as the angle (α) between these trajectories. As before, standard errors on these angles were computed based on the sampling distribution of $\beta^{\mathrm{tr}}$.

Shape changes along the trajectories $\left(\hat{v}\right)$ were visualized from the common reference $\left({\bar{z}}_{a=7}\right)$. Meshes were deformed using thin plate splines and distances between corresponding vertices were reported as colour on the mesh modelling the end of the trajectory between two developmental stages. These visualizations were computed with the Morpho R package [33].

## 3. Results

### 3.1. Allometry and shape variation between species

**Figure 1.**
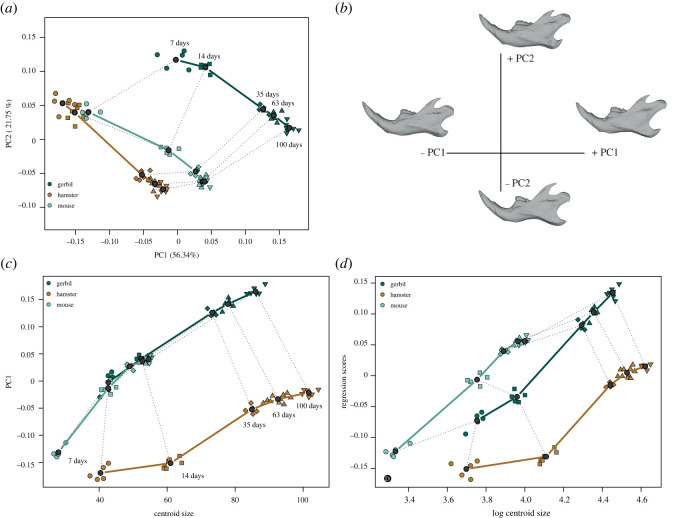
Shape variation of the mandible along the postnatal trajectories. (*a*) First two principal components (PC) of the variation in mandible shapes across ages and taxa. (*b*) Shape changes in relation to PCs based on a mouse reference mesh. (*c*) Relationship between PC1 scores and centroid sizes. (*d*) Regression scores of the allometric model. Dark grey dots represent expected marginal means computed for each age by species group. Dotted lines match equivalent ages across species. Ages are given in days after birth.

The first two components of the principal component analysis (PCA) account for 78.09% of the total shape variance (figure 1*a*). Within species × age variances are much smaller than the ontogenetic variances (electronic supplementary material, table S1), which spread on the two first components. The 7-day-old hamsters and mice are grouped together with the 14-day-old hamsters in the morphospace. Gerbils appear more distant on these first two components in relation to an important shape difference of the 7-day-old juveniles. PC1 is highly related to the allometric vectors (about 37° for gerbils and mice and 46° for hamsters), and corresponds to shape changes with an elongation of the processes, an expansion of all the masseteric fossa, associated with a compression and straightening of the diastema (figure 1*b*). The shape difference of gerbils compared with mice is mainly related to allometry as the 7-day-old gerbils cluster with the 14-day-old mice on PC1 for a similar size (figure 1*c*). Some non-allometric differences remain, as the regression scores of the two species do not overlap (figure 1*d*).

### 3.2. Postnatal developmental trajectories

**Figure 2.**
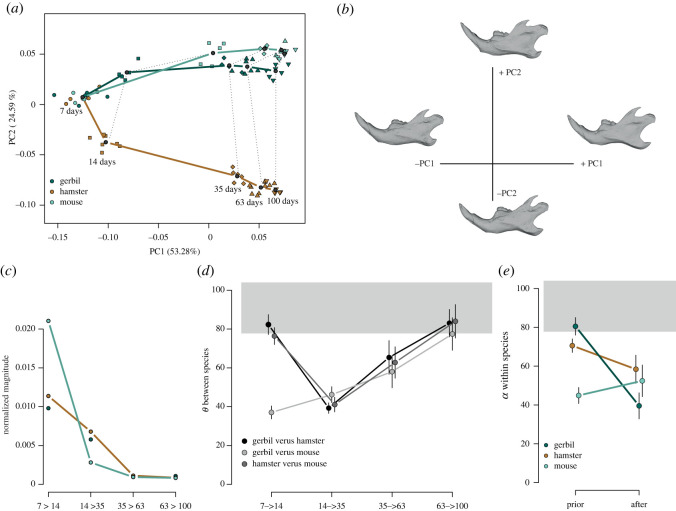
Analyses of postnatal trajectories after parallel transport on the common reference (average of the expected marginal means at 7 days). (*a*) First two parallel transported principal components (PC). (*b*) Shape changes in relation to parallel transported PCs based on mouse reference mesh. (*c*) Magnitude (in Procrustes unit per days) of the growth vectors of shape changes between two consecutive developmental stages normalized by the age differences. (*d*) Angles between growth vectors of two species at the same developmental interval. (*e*) Angles between the 14–35 days vector and the 7–14 days (prior) or the 35–63 days (after) growth vectors for each species. Error bars represent sampled standard errors.

After accounting for shape differences between species using parallel transport, the 7-day-old juveniles are grouped together by design (figure 2*a*), and the trajectories of the two murids (gerbils and mice) traced similar paths, separating from the cricetid (hamsters) on PC2. Thus, gerbils and mice share similar patterns of ontogenetic shape changes (i.e. similar anatomical parts of the mandible are modified in a similar way), whereas hamsters show shape changes along their ontogenetic trajectory different from the two other species (i.e. different parts of the mandible are modified or similar ones but in a different manner). The two murids tend towards an adult shape with a wide masseteric fossa whose ventral ridge is very marked, a short coronoid process, a wide condylar process, as well as a shortened and well-verticalized diastema. Cricetids differentiate during development by acquiring a more horizontalized mandible with a less extensive masseteric fossa than in murids, as well as highly developed angular and coronoid processes, deepening the lunar and the posterior notch. The diastema is more flat and more elongated (figure 2*b*). Description of ontogenetic shape changes per species is available in electronic supplementary material, figure S2.

Overall, the amount of shape changes along the ontogeny is similar between species (path distances equal to 0.26 ± 0.01 for gerbils and mice and 0.28 ± 0.01 for hamsters, electronic supplementary material, table S2). By contrast, the timing of these changes differs between species. A striking pattern is the amount of shape changes in mice between 7 and 14 days compared with the two other species (figure 2*c*). When hamsters and gerbils show a similar rate of shape changes (0.011 and 0.009 per day, respectively), in mice, however, this portion of the trajectory is twice as high (0.021 per day). Rate of shape changes decreases drastically in mice (0.002 per day) between 14 and 35 days, while it stays about threefold higher in hamsters (0.006 per day) and gerbils (0.005 per day). From 35 days, whatever the species, the trajectories show minimal rates (between 0.0009 and 0.002 per day). Thus, whereas hamsters and gerbils have comparable rates of shape changes all along their ontogenetic trajectories (rates that seem to drop linearly until 35 days old), the mice show a different shape of their trajectory (electronic supplementary material, table S3) with a very condensed and rapid shape ontogeny.

As suggested above (figure 2*a*), gerbils and mice share quite similar directions of shape changes along their ontogenies (starting with an angle of 37°), accumulating progressively more differences (figure 2*d*). The average directions of these two trajectories have an angle of 33.6°, but again with a timing shift between these two species. On the contrary, the hamsters have in the first step a very different direction of shape changes (forming an angle of 82.30° with the gerbils and 76.39° with the mice). However, from 14 days old, the directions of shape changes in hamsters are much more similar to the two other species, with a minimal angle for the shape changes arising between 14 and 35 days (*θ* = 39.28° with the gerbils and *θ* = 46.20° with the mice). At this stage, the shape trajectories are in the direction of the common pattern of growth (transported PC1) with angles ranging from 23.7° for hamsters to 33° for gerbils (30.5° for mice, *p* < 0.001), and correspond to the enlargement of the ascending ramus (figure 2*b*). Differences in directions between the hamsters and the two murids accumulate afterwards. The average directions of the three trajectories differ (electronic supplementary material, table S2) but with an angle on average of 36.7° much smaller than expected between two random vectors (*p* < 0.0001).

The directions of shape changes (figure 2*e*) arising within species prior (7–14 days) to the weaning phase (14–35 days) are quite different to the trajectory during weaning in both the hamsters (80.5°) and the gerbils (70.5°). On the contrary, the directions after weaning (35–63 days) are much more similar to the weaning phase with angles varying from 39.5° in hamsters to 58.4° in gerbils. In mice, the pattern of differences is more stable with an angle of about 50°, but again, in mice, the ramus enlargement is more precocious in the postnatal growth.

## 4. Discussion

Our result supports a shape differentiation of early young gerbils partly by allometric scaling compared with mice. Gerbils have a longer gestation of 7 days compared with the other two species [48]. This particularity modifies the onset of postnatal period [49] and explains an already differentiated mandible at birth [50]. After birth, gerbils present a similar general trajectory than the two other species but shifted according to this initial offset. Hamsters and mice, despite a large size difference at birth, share a common mandible shape in the first stage of the postnatal growth.

A common pattern of morphological changes during the postnatal development in the three rodent species studied was observed. This pattern is partly related to the allometry. Indeed, the mandible of juveniles presents small processes that will later develop, allowing the growth of the masseteric fossa. In addition, the diastema, which is initially long and flat, will gradually deepen to allow the incisor to become vertical. This repatterning highlights the strong modification of the posterior part of the mandible during early stages of the postnatal growth. Such changes of the mandible have already been observed in mice [20], in the Sciuridae [21] as well as at a larger scale in the Myomorpha and Hystricomorpha [14]. This geometrical re-spatialization is likely to go hand in hand with the development of the masseter muscles, which allow articulation between the skull and the mandible. The posterior part of the mandible develops to accommodate and resist the pressure induced by the activation of these muscles, allowing the transition from sucking to chewing movement during weaning [51].

The amount of shape change occurring throughout the ontogeny is similar across species according to the similar growth duration and despite very different body mass [52]. However, the timing of these changes, i.e. the relative contribution of each stage to the total amount, differs greatly in mice compared with the two others. In gerbils and hamsters, this timing is quite well conserved with most of the changes occurring equally between 7 and 35 days. In hamsters, the first signs of chewing and masseter muscle action are only observed at 14 days [53], and around 16 days in gerbils [54]. Thereby, the mandible of the mouse undergoes major changes from the first stage (7–14 days). Indeed, it has already been shown that the bite force of mouse juveniles increases well before food weaning [55], which may explain early bone remodelling [56]. Early competition among pups and lower parental care in mice [10,57,58] may explain this acceleration of shape development and the correlated functional changes in mice compared with the two other species.

Gerbils and mice share quite similar directions of shape changes along their ontogenetic trajectories, meaning that similar anatomical parts of the mandible are modified in a similar way, and progressively diverge through the ontogeny. The hamsters have, in the first step, a very different direction of shape changes that seems supported by an important growth of the coronoid process. This differentiation is consistent with the known divergences of the mandibular movement during chewing. It contrasts the mouse and gerbil, which chew with a propalinal motion [59–62], to the hamster, which exhibits an oblique motion [59,61]. Anatomical constraints on the jaw movements appear to be established at an early age and may therefore also influence sucking movements. This early onset of specific movements could in turn influence bone growth as human-induced changes do [60]. However, movements may not be strictly constrained and, for instance, propalinal movements have been observed in hamsters to adapt their chewing to seed processing [63]. Ontogenetic data on the acquisition process of movement types are therefore needed to reach any robust conclusion but are not yet documented to our knowledge.

After these early anatomical differences, the ontogenetic trajectories of the three species become more similar between 14 and 35 days. This channelling of the ontogenetic variation corresponds to shape changes in the direction of the main pattern of changes (parallel transported PC1), which appears to be related to the expansion of the ascending ramus. The transition of mandible movement between sucking and chewing occurs at around 21 days for mice and hamsters [64,65] and 30 days for gerbils [48]. This transition implies new mechanical constraints on the bone [66], which may explain the convergence of the directions of change at this stage between the three species. If the early divergence of the trajectories relates to the movement acquisition, then their convergence at weaning is probably more related to the functional load of the mandible by muscles than to the movements themselves. Reduction in muscle mass or activity by the use of soft diet, chirurgical intervention or in the case of muscular dystrophy led to smaller condyle and reduction in the height of the ramus [67] in agreement with the observed pattern of shape changes.

In mice, the directions of shape changes before and after weaning are just as different from the changes that occur during the weaning phase. Indeed, the intense shape changes occurring before 14 days in this species may explain this similarity. In hamsters and gerbils, the directions of shape changes within species prior to the weaning are quite different to the trajectory during weaning. As expected, if the mechanical forces induced by mastication influence growth [68,69], the directions after weaning are much more similar to the direction of shape changes during the weaning phase for these two species than the prior-weaning trajectories. Thus, in hamsters and gerbils, the weaning period seems to have a significant impact on shape ontogeny in implying similar local bone growth in relation to the functional constraints. The mechanical stresses of occlusion and masticatory function during this period implied an adaptive response of the bone both in term of anatomy [10] and structure [60,70]. Changes in feeding behaviour are associated with changes in social behaviour (behavioural weaning) with the mother and other juveniles [61,62,71]. Fighting or playing with siblings for example [24] will also contribute to the total physical stress applied to the mandible and therefore will contribute to the effect of bone loading on shape changes occurring at this period. This bone response at both structural and anatomical levels could allow an indirect response to some potential selection arising at this stage among siblings and conspecifics. This indirect process may then have driven some mandibular shape divergences over time.

## 5. Conclusion

Throughout the postnatal ontogeny, similar shape changes, with an increase in the posterior part of the bone and a straightening of the diastema, are observed across the three rodent species. Nonetheless, mice present very different timing compared with the gerbils or the hamsters. The mouse mandible undergoes most of its changes just before weaning, thus inducing little shape modifications during and especially after this event, which may be related to the early increase of bite force observed in the literature. On the other hand, the longer gestational period of the gerbil seems to condition a time-shifted trajectory and a mandibular shape divergence partly by allometric scaling in comparison with the mouse. After an early shape divergence between subfamilies, muscular loading and other mechanical stresses arising around weaning drive similarly the ontogenetic shape changes in both gerbils and hamsters, consistent with the earlier changes observed in mice.

## Data Availability

Data are available at the Dryad Digital Repository (https://doi.org/10.5061/dryad.ffbg79cx3) [72]. All analytical codes are available in R packages or open software Slicer. The data are provided in electronic supplementary material [73].
